# Structure-Based Modeling of Complement C4 Mediated Neutralization of Adenovirus

**DOI:** 10.3390/v13010111

**Published:** 2021-01-15

**Authors:** Corey C. Emerson, Phoebe L. Stewart

**Affiliations:** Department of Pharmacology and Cleveland Center for Membrane and Structural Biology, Case Western Reserve University, Cleveland, OH 44106, USA; cxe81@case.edu

**Keywords:** adenovirus, neutralization, neutralizing antibody, complement C1, complement C4, molecular dynamics

## Abstract

Adenovirus (AdV) infection elicits a strong immune response with the production of neutralizing antibodies and opsonization by complement and coagulation factors. One anti-hexon neutralizing antibody, called 9C12, is known to activate the complement cascade, resulting in the deposition of complement component C4b on the capsid, and the neutralization of the virus. The mechanism of AdV neutralization by C4b is independent of downstream complement proteins and involves the blockage of the release of protein VI, which is required for viral escape from the endosome. To investigate the structural basis underlying how C4b blocks the uncoating of AdV, we built a model for the complex of human adenovirus type-5 (HAdV5) with 9C12, together with complement components C1 and C4b. This model positions C4b near the Arg-Gly-Asp (RGD) loops of the penton base. There are multiple amino acids in the RGD loop that might serve as covalent binding sites for the reactive thioester of C4b. Molecular dynamics simulations with a multimeric penton base and C4b indicated that stabilizing interactions may form between C4b and multiple RGD loops. We propose that C4b deposition on one RGD loop leads to the entanglement of C4b with additional RGD loops on the same penton base multimer and that this entanglement blocks AdV uncoating.

## 1. Introduction

There are multiple parallel pathways for neutralizing pathogens such as adenovirus (AdV). While neutralization pathways are beneficial in the case of natural infections, they represent roadblocks in the development of virus-based therapeutics, such as oncolytic viruses [1], and gene therapy vectors [2,3]. Both pre-clinical and clinical data showed that anti-AdV-specific neutralizing immunity reduce efficacy of AdV-based vaccines, including against HIV-1 [4], and SARS-CoV-2 [5]. Therefore, a better understanding of the molecular mechanisms underlying host neutralization pathways, specifically involving neutralizing antibodies and complement, would be beneficial for engineering AdV-based therapeutics with improved safety and efficacy. 

Following AdV infection, both the innate and adaptive arms of the immune system are involved in the clearance of the virus. When human species C HAdV-C5 is injected into the bloodstream, the innate immune system responds with natural immunoglobulin M (IgM) antibodies [6,7,8], and coagulation factor X (FX) [9,10], to opsonize the virus and target it for clearance. For HAdV-C5, natural IgM binds to the hypervariable region 1 (HVR1) of hexon, the major capsid protein, which forms a repetitive, negatively charged pattern on the capsid surface [11]. IgM binding to AdV activates the complement cascade, leading to the covalent binding of first complement component C4b and then C3b to the virus [12]. The blood coagulation factor, FX, binds species C HAdV-C2 and HAdV-C5 with high affinity via the major capsid protein, hexon, and helps to target the virus to the liver for clearance [9,10]. Effectively, the FX-decorated surface of AdV becomes a pathogen-associated molecular pattern (PAMP), which, after internalization into a macrophage cell, serves to activate innate immunity via the TLF/NF-κB pathway [13]. The binding of IgM and FX to AdV represent parallel host–virus neutralization pathways, as FX binding to AdV protects the virus from complement-mediated inactivation [12].

During the initial exposure to a particular virus, innate immune responses activate and stimulate adaptive immune responses, which are ultimately responsible for complete viral clearance [14]. Adaptive immunity includes both a humoral immune response, involving B cells and CD4 helper T cells, and a cell-mediated immune response, involving CD8+ T cells. B cells produce virus-specific antibodies that can neutralize and inactivate virions. Virus-specific immunoglobulin G (IgG) antibodies, similar to IgM, can activate the complement system after binding to a virus particle [15]. Complement proteins serve to opsonize pathogens and induce inflammatory responses that help fight infection. The complement system is an integral effector part of both the innate and adaptive immune response to viral infections. 

The classical pathway of complement activation begins with the binding of the C1 complex (C1q, C1r_2_, C1s_2_) to antigen-bound IgM or IgG [15]. IgM exists in circulation as planar pentameric and hexameric assemblies with its C1q binding site hidden [16]. After antigen binding, a conformational change occurs in IgM to convert it into a staple-like conformation with exposed C1q binding sites [17,18,19]. Only one antigen-bound IgM is needed to activate complement, whereas several IgG molecules bound to the antigen in close proximity are required for activation [16]. Several studies have shown that IgG antibodies oligomerize and form platforms with their F_C_ domains to present appropriately spaced C1q binding sites [20,21,22]. C1q is a hexamer formed by heterotrimeric chains A, B and C, assembled into a bundle of six collagen helices and six globular recognition domains that bind immunoglobins [23]. C1r and C1s are both serine proteases. After the C1q globular domains interact with antigen-bound IgM or IgG, C1r is activated, which in turn activates C1s [16]. Activated C1s cleaves complement component C4 into C4a, which is released, and C4b, which has a highly reactive thioester that can react with hydroxyl or amino groups near the antibody binding site on the pathogen. The classical pathway continues with an enzyme cascade involving complement components C2 and C3. Like C4b, C3b has a reactive thioester that can opsonize the pathogen and C3b opsonization is involved in complement signal amplification [15]. 

Recently, Bottermann et al. have shown that, after an anti-hexon neutralizing IgG, called 9C12, binds HAdV-C5, complement components C1 and C4 mediate the potent neutralization of HAdV-C5 and that this antiviral activity is independent of downstream complement components [24]. The binding of C4b to HAdV-C5 does not block cell entry, but rather blocks the release of the virally encapsidated membrane lytic factor, protein VI, which is essential for escape from the endosome [25,26]. The antiviral mechanism of C4 is reminiscent of AdV neutralization by α-defensins, which are proteins of the innate immune system that suppress viral and bacterial infections [27]. Human α-defensin 5 (HD5) has potent antiviral activities against many but not all human AdV types [28]. HD5 binds at the interface between the AdV penton base and the fiber capsid proteins, stabilizes the vertex region of sensitive AdV types, and prevents the release of protein VI [28,29].

A cryo-electron microscopy (cryo-EM) study of antigen-bound IgG in complex with C1 revealed that bound IgG’s adopt a bent conformation and form a hexameric F_C_ platform, which binds four, five or six C1q globular domains [22]. The moderate resolution cryo-EM structure of the complex, combined with atomic resolution structures of the C1q globular domain [30], and IgG F_C_ domains [31], facilitated the generation of a structural model for C1q-IgG interaction. A cryo-electron tomography (cryo-ET) study of IgM, C1, and C4b complexes formed on antigen-bearing lipid membranes revealed pentameric and hexameric IgM complexes in dome-shape conformations with bound C1 and C4 [19]. These IgG-C1 and IgM-C1 complex structures reveal similarly spaced C1q binding sites of the periphery of their respective F_C_ platforms. The structure of the IgM complex also revealed additional density for C4b oriented next to the IgM-Fab arms. The thioester-containing domain of C4b is positioned such that the reactive thioester points toward the antigenic surface. 

In this study, we used structural information on the binding of the IgG 9C12 to HAdV-C5 [32,33], together with cryo-EM and cryo-ET structures of IgG-C1 and IgM-C1-C4 complexes [19,22], to build a composite model for HAdV-C5 with bound IgG, C1, and C4b. The goals of this study were to evaluate likely C4b binding sites on the HAdV-C5 capsid and investigate the structural mechanisms underlying the C4b neutralization of HAdV-C5. A prior cryo-EM structure of HAdV-C5 with 9C12 IgG molecules indicated strong density for 9C12 bound to the peripentonal hexons, which are the five hexons surrounding the penton base capsid protein at the vertices of the AdV capsid [33]. The pentameric penton base has five intrinsically disordered Arg-Gly-Asp (RGD)-containing loops that protrude from penton base and interact with αv integrins on host cells triggering internalization of the virus [29,34,35]. The composite model we built for HAdV-C5 with IgG 9C12, C1 and C4b indicated that C4b might bind to various solvent accessible hydroxyl and amino groups within the penton base RGD loop. We performed molecular dynamics simulations with C4b covalently bound to two possible sites within the RGD loop. The results of these simulations indicate that C4b binding to one RGD loop of a HAdV-C5 penton base will likely result in additional stabilizing interactions between C4b and another RGD loop of penton base. In addition, since the highly reactive thioester of C4b can react with a water molecule before reaching the pathogen [15], we also performed a molecular dynamics simulation with C4b positioned near, but not covalently bound to, the penton base RGD loops. This simulation indicates that, when C4b is positioned near one RGD loop, even without being covalently bound to the penton base, C4b may form additional stabilizing interactions with nearby RGD loops. This work revealed alternate mechanisms of how C4b might block AdV uncoating by entangling the RGD loops of the penton base with or without covalent binding to the virus and suggests strategies that might be used to modulate the interaction of AdV with the complement system. Our computational modeling-based analyses may prove useful in designing future biological experiments to evaluate complement-AdV interactions through the introduction of targeted mutations in the AdV capsid to reduce virus sensitivity to complement and, thus, aid in designing therapeutic vectors resistant to complement-mediated neutralization.

## 2. Methods

### 2.1. Model Building

For the penton base, Rosetta-based models for the RGD loop aa 297–376 [29] were added to the cryo-EM HAdV-C5 penton base coordinates (PDB: 6B1T) [36]. Five different RGD loop models were used, one for each subunit of the penton base pentamer (chains A–E). One icosahedral facet of hexons (12 trimers), plus two edge hexons from each of the three adjacent facets (6 additional trimers), were selected from the cryo-EM HAdV-C5 structure (PDB: 6B1T) with UCSF ChimeraX v1.1 [37]. Three penton base pentamers with modeled RGD loops were added to the adjacent vertex sites to form a model of the HAdV-C5 facet. Coordinates for 9C12 Fab fragments were positioned above hexon epitopes by aligning the crystal structure of the hexon in complex with the 9C12 Fab (PDB: 5LDN) with each hexon subunit in the HAdV-C5 facet [32]. The UCSF Chimera v1.15 MatchMaker tool was used for alignment [38]. The partially occupied facet/Fab model, with two thirds of possible hexon epitopes occupied with 9C12 Fab, was generated by removing Fab fragments from the fully occupied facet/Fab model with UCSF Chimera v1.15. Fab fragments were selected for removal to minimize steric clashes between Fab fragments and to approximate the Fab density observed in the cryo-EM structure of HAdV-C5 with 9C12 IgG [33].

To build a model of an HAdV-C5 facet with a hexameric IgG F_C_ platform, the F_C_ platform coordinates from a cryo-EM structure of an IgG-C1 complex (PDB: 6FCZ) were used [22]. The hinge region of one F_C_ was positioned near the exposed CL, CH_1_ domains of a 9C12 Fab fragment positioned on a peripentonal hexon. The additional five hinge regions of the hexameric F_C_ platform were positioned more approximately over other Fab fragments in the partially occupied facet/Fab model. The hexameric F_C_ platform positioned over the HAdV-C5 facet was used as a guide to add in coordinates for six C1q globular domains (PDB: 6FCZ) and the cryo-EM density for IgG-C1 (EMD-4232) [22].

Given the strong similarity between the cryo-EM structure of an IgG-C1 complex and the cryo-ET structure of an IgM-C1-C4 complex [19,22], we used the position of C4b in the later structure to guide the positioning of C4b relative to the C1 complex modeled with HAdV-C5. Coordinates from the crystal structure of C4b (PDB: 4XAM) were used to complete the HAdV-C5/9C12/C1/C4b model [39]. All graphic figures were prepared with UCSF Chimera v1.15 [38]. 

### 2.2. Molecular Dynamics Simulations

A molecular dynamics simulation was performed to assess the solvent accessibility of possible C4b opsonization sites within the penton base RGD loop. A simulation for the penton base pentamer with Rosetta-based RGD loop models was performed with NAMD v2.12 on the Case Western Reserve University (CWRU) high-performance computing (HPC) cluster [40]. The molecular system was minimized for 50 ps, followed by slow heating to 300 K. A molecular dynamics simulation was run for 5 ns using the Chemistry at Harvard Molecular Mechanics (CHARMM) force field [41], with Generalized Born implicit solvent (GBIS). The solvent accessibility of the atoms in the hydroxyl groups of serines and threonines, and the amino groups of lysines and arginines, within the five RGD loops was assessed for the starting and ending coordinates with the UCSF ChimeraX v1.1 “measure sasa” command and a probe radius of 1.4 Å [37]. 

Molecular dynamics simulations were performed to assess the possibility that C4b would interact with multiple RGD loops on one penton base pentamer. The coordinates used for C4b were from the crystal structure (PDB: 4XAM) [39]. Six different starting models for C4b relative to a penton base pentamer were prepared for molecular dynamics simulations. Four of the starting models (models 1, 2, 4, and 5) were generated with a covalent bond between Cys1010 of C4b and a residue within one penton base RGD loop (Thr343, chain C; Arg347, chain C; Thr346, chain C; or Lys297, chain E, respectively). We used UCSF Chimera v1.13 to prepare the chosen RGD loop residue, position the sulfur atom of the reactive thioester on C4b near the appropriate atom of the RGD loop residue, form a covalent bond with the “bond sel” command, and change the chain IDs to be the same for the two covalently linked polypeptides [38]. Two starting models (models 3 and 6) were generated without a covalent bond between C4b and the penton base. For these models, the Cys1010 of C4b was positioned ~10 Å from one RGD loop of the penton base (chain C or chain E, respectively). Molecular dynamics simulations were performed with NAMD v2.12 on the Case Western Reserve University (CWRU) high-performance computing (HPC) cluster [40]. The molecular systems were minimized for 50 ps followed by slow heating to 300 K. Molecular dynamics simulations were run for 12 ns using the Chemistry at Harvard Molecular Mechanics (CHARMM) force field [41] with generalized born implicit solvent (GBIS).

### 2.3. Calculation of Non-Bonded Interaction Energies

Non-bonded interaction energies, including van der Waals and electrostatic components, were calculated between C4b and each chain of the penton base individually, as well as between C4b and the penton base multimer (chains A–E) as a whole. The energy calculations were performed for both starting and ending coordinates of the molecular dynamics simulations of models 1–6. NAMD v.2.14 [40] and the NAMD Energy plugin of VMD v1.9.3 [42], both running on a Windows 10 PC, were used to calculate the interaction energies.

## 3. Results

### 3.1. Modeling of HAdV-C5 with Antihexon Neutralizing Antibody

**Figure 1:**
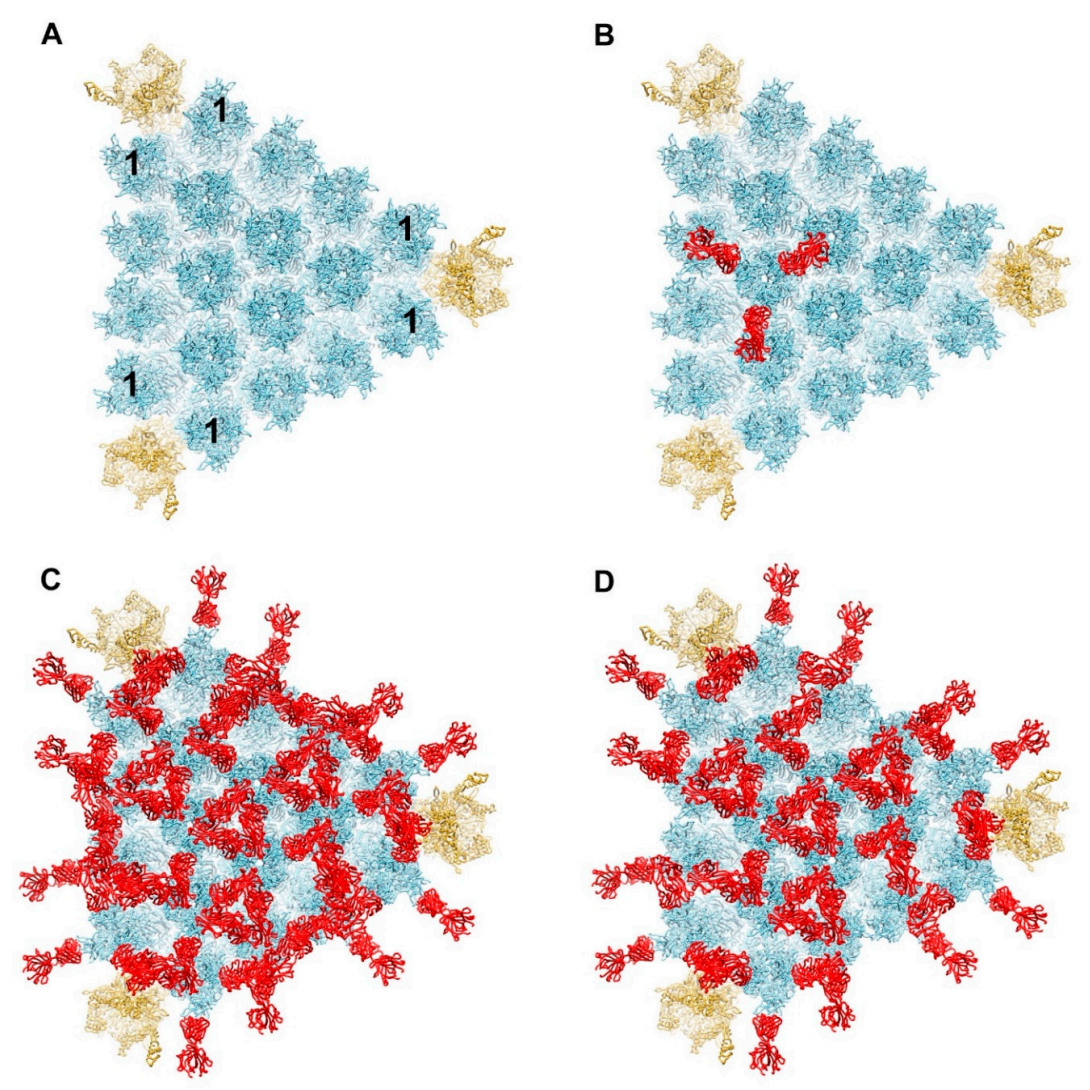
Model for binding of antihexon monoclonal antibody 9C12 to a facet of HAdV-C5. (**A**) Major capsid proteins in one facet of HAdV-C5 with hexons forming an array in the middle (light blue) and penton bases at the vertices (gold) (PDB: 6B1T) [36]. The peripentonal hexons are denoted with the number 1. Each penton base is shown with Rosetta-based models for the RGD loops [29]. (**B**) One hexon trimer shown with three 9C12 Fab fragments (red) positioned as in the crystal structure of isolated hexon with 9C12 Fab (PDB: 5LDN) [32]. (**C**) Fully occupied model of HAdV-C5 facet with all hexon epitopes occupied with a 9C12 Fab. (**D**) Partially occupied model with two thirds of the possible hexon epitopes occupied with a 9C12 Fab. The occupied Fab binding sites were selected so that the model would resemble the cryo-EM structure of HAdV-C5 with 9C12 IgG [33].

Previous work has shown that a particular anti-hexon neutralizing IgG, called 9C12, stimulates binding of complement component C4b to the capsid and mediates potent neutralization of HAdV-C5 [24]. Although there is both cryo-EM and crystallographic structural information on the binding of 9C12 to HAdV-C5 [32,33], there are still open questions about how this particular IgG interacts with the full virion. It has been shown that the minimum ratio of 9C12 to HAdV-C5 for neutralization is 240 antibody molecules per virus particle, which is equivalent to an average of two Fab fragments per hexon trimer [33]. In other words, assuming IgG binds bivalently, only two thirds of the available epitopes need to be occupied to achieve neutralization. The crystal structure of the isolated HAdV-C5 hexon with 9C12 Fab fragments revealed that the epitope includes hexon hypervariable regions (HVRs) 2 and 8, which form the outer corner of each of the three towers of a hexon trimer [32]. The cryo-EM structure of HAdV-C5 with intact 9C12 indicated bivalent binding for the IgG [33]. Strong density was observed for Fab fragments on the peripentonal hexons, hexons adjacent to penton base (Figure 1A), as well as a meshwork of Fab density covering the rest of the hexon capsid surface. The cryo-EM density was interpreted as indicating 100% occupancy of Fab at the peripentonal hexon sites and a spatial average of many alternate bivalent binding combinations for 9C12 on the rest of the capsid. 

We built a model for the interaction of 9C12 Fabs with one facet of HAdV-C5 based on the available cryo-EM and crystallographic structural information. Initially we positioned three Fab fragments on each hexon trimer in the facet, as indicated by the crystal structure (Figure 1B). This resulted in a fully occupied model with multiple clashes between Fab fragments (Figure 1C). Clashes between Fab fragments, as well as the consideration of expected steric hindrance between IgG F_C_ fragments, led us to conclude that the fully occupied model is unrealistic. Therefore, we reduced the number of Fab fragments bound to one facet so that two thirds of the possible epitopes were occupied with a 9C12 Fab (Figure 1D). This partially occupied model displays fewer steric hindrances between neighboring IgG Fab and F_C_ fragments and better resembles the cryo-EM structure of the HAdV-C5/9C12 complex [33]. There is undoubtedly variation in how the 9C12 antibody binds to hexons in each icosahedral facet and in each virus particle. Therefore, the model shown in Figure 1D is meant only to be a representative approximation. In building the partially occupied model, we left all three epitopes on each peripentonal hexon occupied with Fabs in accordance with the strong cryo-EM density observed at these sites. 

**Figure 2:**
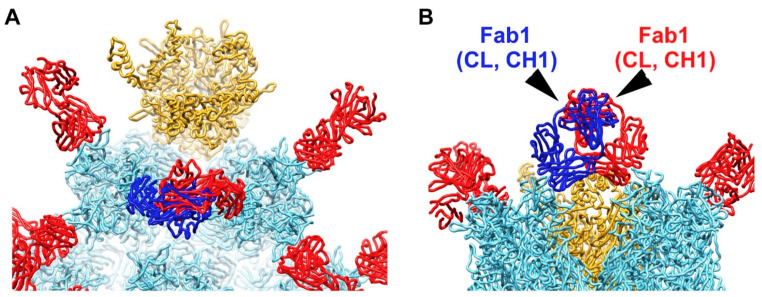
Steric clashes are modeled between two 9C12 Fab fragments bound to neighboring peripentonal hexons. (**A**) Enlarged view of vertex region with two hexon trimers and one penton base with selected 9C12 Fab fragments as in Figure 1D. A severe steric clash is modeled between Fab fragments (blue and red) bound to neighboring peripentonal hexons. (**B**) Perpendicular view of panel A. The clashing domains (CL, CH_1_) of each Fab are indicated.

As a result of leaving all peripentonal hexon epitopes occupied with a Fab in the partially occupied model, major steric clashes are observed between the CL and CH_1_ domains of two Fabs (Figure 2). However, it was noted in the cryo-EM structure of the HAdV-C5/9C12 complex that the IgG density at this site had one well-shaped Fab arm and one somewhat distorted Fab arm [33]. The cryo-EM structure indicated that the binding of 9C12 to these two peripentonal epitopes resulted in an unusually acute angle between the long axes of the Fab fragments. Varghese et al. concluded that bivalent binding of 9C12 to this site was likely facilitated by the inherent segmental flexibility of IgG molecules [33]. In addition, we note that the crystal structure of the isolated hexon with the 9C12 Fab reveals the epitope to be composed mainly of two HVR regions [32]. We suspect that the conformational flexibility of the epitope region might contribute to the bivalent binding of 9C12 to this apparently strained IgG binding site. Indeed, Bottermann et al. note that 9C12 does not display a particularly fast on-rate with hexon and that this observation might be explained by an entropic cost associated with engaging a structurally variable epitope [32]. 

### 3.2. Modeling of HAdV-C5 with IgG and Complement Components C1 and C4b

The cryo-EM structure of the HAdV-C5/9C12 complex did not reveal defined density for the F_C_ regions, indicating variability in the F_C_ positions relative to the HAdV-C5 capsid [33]. The lack of observed F_C_ density in the cryo-EM structure of the HAdV-C5/IgG complex is not surprising given the known flexibility of IgG molecules [43]. The antihexon antibody 9C12 is of the IgG1 subclass of antibodies. Extensive structural flexibility has been observed for IgG1 molecules by individual-particle electron tomography 3D reconstruction [44]. The partially occupied Fab model shown in Figure 1D does not include modeled F_C_ regions for the bound 9C12 IgG molecules. However, modeling the locations of the 9C12 IgG F_C_ regions is important for adding complement components C1 and C4b to the HAdV-C5/9C12 model, since the F_C_ regions contain binding sites for the globular recognition domains of C1q [43]. The C1q binding site is near the IgG hinge region and is thought to be partially or completed shielded by the Fab arms when IgG is not bound to an antigen [43]. It has been suggested that the F_C_ regions of multiple IgG molecules form hexamers when opsonized on target surfaces [21]. Mutations can be introduced in IgG that drive the formation of IgG hexamers in solution [20,21,45]. The cryo-EM structure of a soluble C1-IgG complex was formed with hexamer-promoting IgG molecules [22]. The docking of the cryo-EM density for the soluble C1–IgG complex with known atomic resolution structures of the component domains resulted in the identification of the C1q binding residues within the two F_C_ CH2 domains of an IgG. These C1q binding residues were corroborated with mutagenesis studies. The cryo-EM and cryo-ET structures of IgG-C1 and IgM–C1–C4 complexes indicate that the Fab arms of an IgG hexamer and IgM fold so that they are nearly perpendicular to their respective F_C_ region when C1 is bound to the complex [19,22]. 

**Figure 3:**
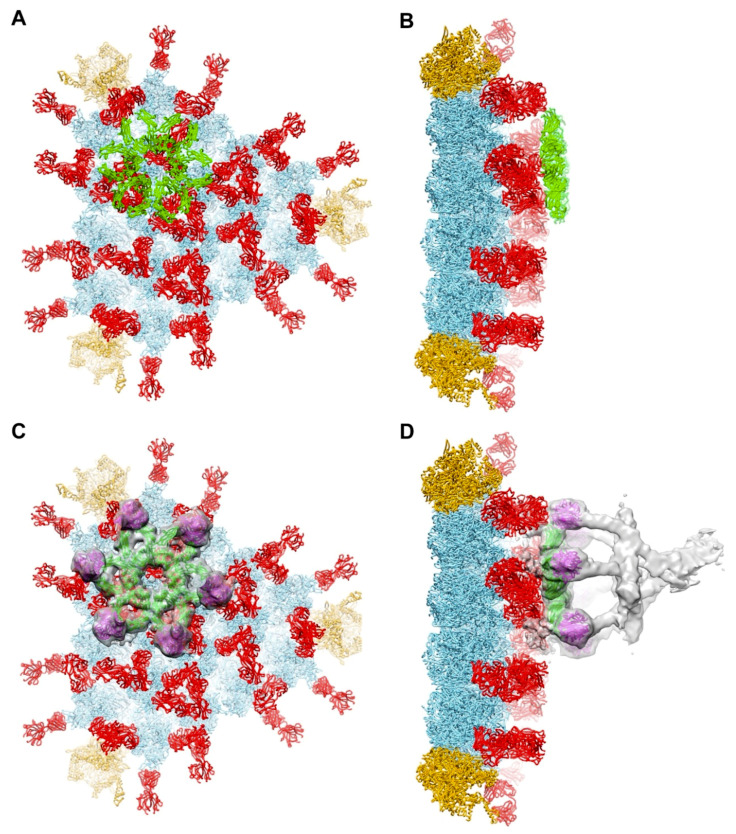
Model for binding of complement C1 complex to a facet of HAdV-C5. (**A**) One facet with selected 9C12 Fab fragments, as in Figure 1D, modeled with a hexameric F_C_ platform (green) from a cryo-EM structure of an IgG-C1 complex (PDB: 6FCZ) [22]. (**B**) Perpendicular view of panel A showing that the F_C_ platform is positioned just above the layer of Fab fragments (red) bound to the hexons. (**C**) One facet, as in panel A, modeled with six C1q globular domains (magenta) from the IgG-C1 structure, shown with a slab of cryo-EM density (transparent gray). (**D**) Perpendicular view of panel C showing the full cryo-EM density map for IgG-C1 (EMD-4232) [22].

In the HAdV-C5/9C12 model, we added a hexamer of F_C_ domains with one IgG hinge region near the Fabs bound to the peripentonal hexons. A preference for 9C12 binding to the peripentonal hexons was noted in the cryo-EM structure of the HAdV-C5/9C12 complex [33]. The other IgG hinge regions of the F_C_ hexamer were positioned roughly near other Fabs bound to hexons in the facet (Figure 3A,B). It was not possible to align the additional five hinge regions of the F_C_ hexamer with particular Fab fragments without distorting the underlying hexon epitopes, bound Fab fragments, or the hexameric FC platform coordinates. Therefore, the FC portion of the HAdV-C5/9C12 IgG model shown in Figure 3 is likely more hexameric than can exist in reality. This is in accord with the lack of a clear hexameric pattern of Fab arms in the partially occupied model of Fabs bound to one facet (Figure 1D). Additionally, we noted that the cryo-EM structure of the HAdV-C5/9C12 complex indicates heterogeneity of occupied Fab binding sites in the middle of the facet [33]. Therefore, we suspect that, in reality, perhaps only four or five F_C_ domains assemble over each HAdV-C5 facet to form imperfect F_C_ hexamers. Nevertheless, the imperfect F_C_ hexamers may still attract the binding of the complement C1 complex if the IgG molecules are bent and if the spacing of C1q binding sites is appropriate. Ugurlar found, in their cryo-EM analysis of soluble C1-IgG complexes, that classification results in separate classes with four, five or six globular C1q domains in contact with F_C_ platforms [22]. Their soluble C1-IgG complexes were formed with IgG molecules mutated to induce hexamer formation, which was undoubtedly useful for structural analysis but which may not represent all F_C_ assemblies that can bind C1. We propose that, with native IgG molecules, such as 9C12, perhaps F_C_ aggregation does not need to form perfect hexamers to induce C1 binding. 

**Figure 4:**
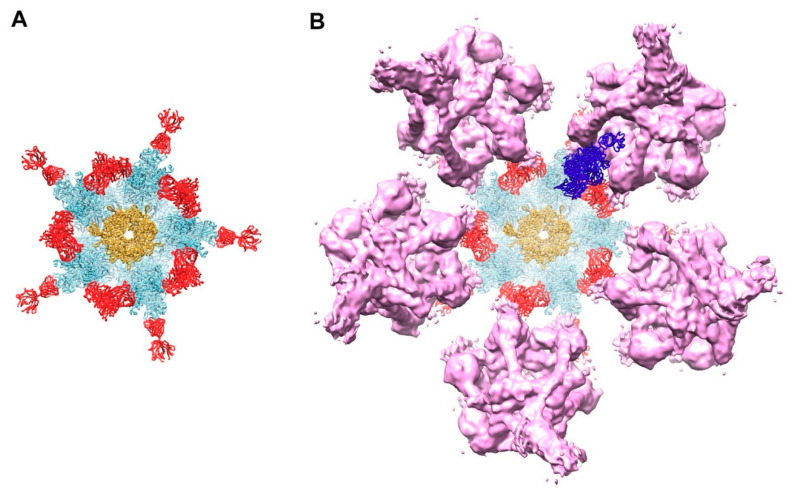
Model for complement C4b near penton base of HAdV-C5 vertex. (**A**) Capsid vertex with a central penton base (gold) surrounded by five peripentonal hexons (light blue) shown with modeled 9C12 Fab fragments (red). (**B**) Capsid vertex with 9C12 Fab fragments modeled with five copies of the IgG-C1 complex (purple) (EMD-4232) [22] near the peripentonal hexons and one copy of complement C4b (blue) (PDB: 4XAM) [39] positioned approximately based on the IgM-C1-C4 structure [19].

With an F_C_ hexamer in the HAdV-C5/9C12 model, it was possible to add in models for six C1q globular domains and cryo-EM density for the soluble IgG-C1 complex (Figure 3C,D). In reality, we expect that the assembly of HAdV-C5/9C12/C1 is more heterogeneous in nature with variations in the F_C_ aggregates. The key factors that the model shown in Figure 3 revealed are (1) that 9C12 IgG molecules bound bivalently to the peripentonal hexons may form F_C_ interactions with other 9C12 IgG molecules bound to the array of hexons in the middle of the HAdV-C5 facet, and (2) that the C1 complex may bind preferentially to the corners of the HAdV-C5 facets near the penton bases (Figure 3C), rather than to the middle of a HAdV-C5 facet. Once a model for HAdV-C5/9C12/C1 was built, it was possible to add in a molecule of complement C4b (Figure 4). We show C4b positioned over a peripentonal hexon, putting C4b in close proximity to a penton base. This position is based on the location of density for C4b in the cryo-ET structures of IgM–C1–C4b complexes [19]. In these structures, Sharp et al. detected density for one or two C4b molecules per complex and found C4b positioned next to Fab arms of IgM in a bent conformation. We admit that, in building the HAdV-C5/9C12/C1/C4b model shown in Figure 4B, we chose to position C4b close to the penton base, when in reality C4b might equally well be located over the middle of the facet. However, as C1 complexes preferentially bind near peripentonal hexons, our model predicts that at least some of the bound C1 complexes would present C4b molecules near a penton base.

### 3.3. Possible Covalent Binding Sites for C4b on HAdV-C5 Penton Base

After recognition of a pathogen by IgG or IgM and recruitment of the C1 complex, the activated C1s serine protease in the C1 complex cleaves complement protein C4 into a C4a fragment (9k Da), which is released into the solvent, and C4b (195 kDa), which acts as an opsonizing factor. C4b has an internal thioester bond that is exposed after conformational changes induced after its cleavage by C1s [39,46]. The thioester of C4b is highly reactive and rapidly forms a covalent bond with a nearby hydroxyl or amino group [15,16]. Often the C4b thioester forms a covalent bond with the surface of the pathogen by interacting with hydroxyl or amino groups, but it can also react with nearby water molecules. Our model for HAdV-C5/9C12/C1/C4b indicates that at least some molecules of C4b will be near the penton base of HAdV-C5 and that the reactive thioester will be oriented toward the penton base (Figure 4B). 

**Figure 5:**
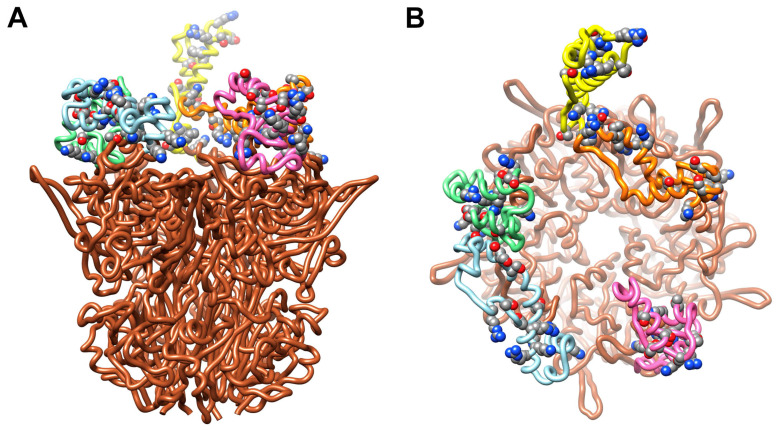
HAdV-C5 penton base with Rosetta-based models for the RGD loops. (**A**) Side view of pentameric penton base (PDB: 6B1T) [36] with different RGD loop models (aa297–376) for each of the five subunits (chain A, pink; chain B, light blue; chain C, green; chain D, yellow; chain E, orange) [29]. All of the sidechains in the RGD loop that contain hydroxyl or amino groups (serines, threonines, lysines and arginines) and that might serve as opsonization sites for the reactive thioester of C4b are shown in space filling representation. (**B**) Top view of panel A.

The cryo-EM structure of HAdV-C5 and the crystal structure of HAdV-C2 penton base both indicate that the integrin-interacting, RGD-containing loops of the penton base are flexible [36,47]. In the HAdV-C5 penton base structure, over 80aa (aa297–376) are missing in the RGD loop due to flexibility and predicted intrinsic disorder [29]. Flatt et al. built Rosetta-based models for the HAdV-C5 RGD loops, which extend ~50 Å above the top of the ordered portion of penton base (Figure 5A) [29]. Intrinsic disorder within the RGD loops may provide a functional advantage for interaction with αv integrins, which serve as internalization receptors for HAdV [35]. The flexible and extended nature of the penton base RGD loops may also make them likely targets for C4b opsonization. Examination of the HAdV-C5 penton base RGD loop sequence indicates eight residues with a hydroxyl group in their sidechain (serines and threonines) and eight residues an amino group (lysines and arginines), all of which might serve as binding sites for the reactive thioester of C4b (Figure 5). A molecular dynamics simulation of the HAdV-C5 penton base pentamer with modeled RGD loops, combined with a calculation of the solvent accessible surface area for the hydroxyl and amino groups, indicated that these possible reactive thioester binding sites are all solvent accessible. The maximum solvent accessible surface area was found for all of these groups in both the starting and ending coordinates (528 Å^2^ for hydroxyl oxygens; 575 Å^2^ for amino nitrogens).

### 3.4. Molecular Dynamics Simulations with HAdV-C5 Penton Base and C4b

**Figure 6:**
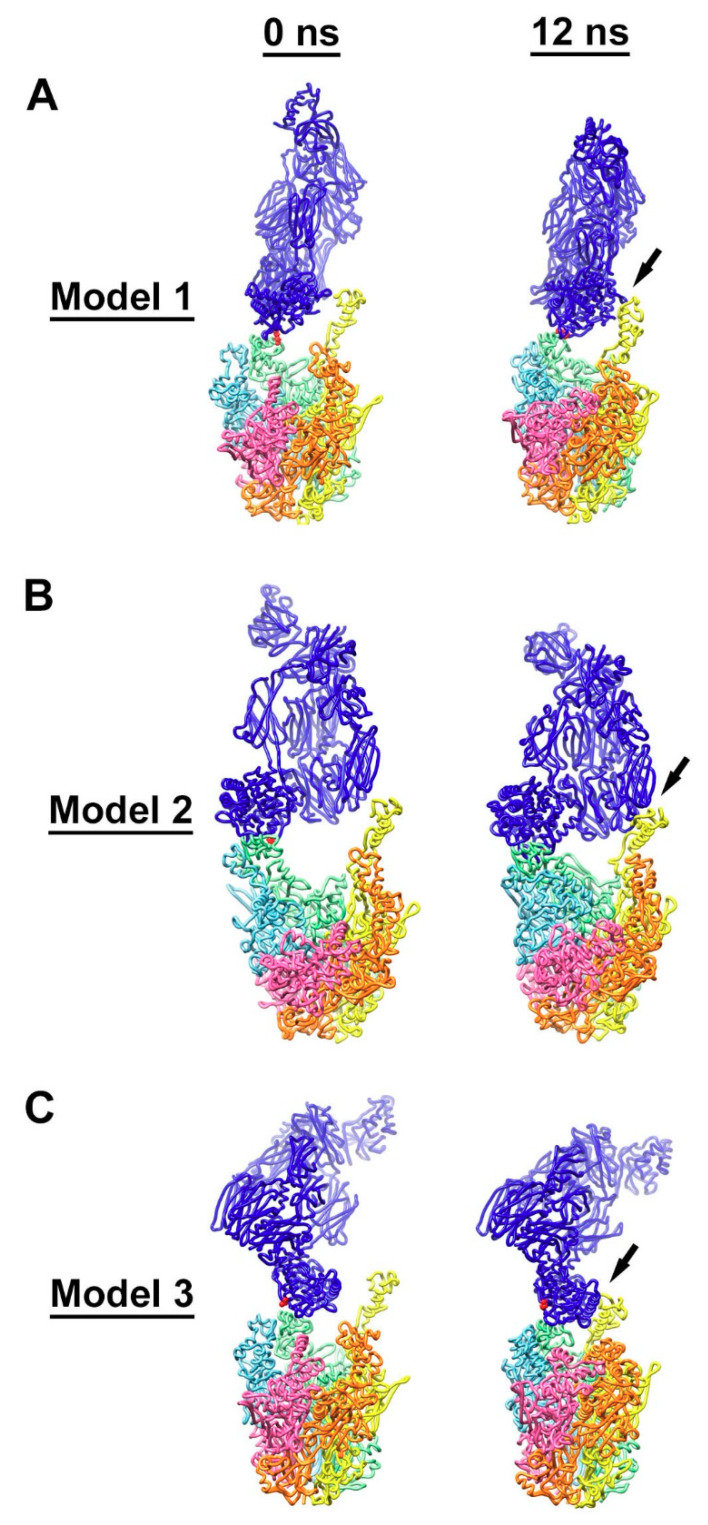
Interaction of C4b with multiple RGD loops of HAdV-C5 penton base. (**A**) Initial (0 ns) and final (12 ns) coordinates from the model 1 molecular dynamics simulation with a covalent bond between Thr343 of penton base chain C (green) and C4b (blue). The covalently linked residues, Thr343 of penton base and Cys1010 of C4b, are in red. (**B**) Coordinates from the model 2 simulation with a covalent bond between Arg347 of penton base chain C (green) and C4b. The covalently linked residues, Arg347 of penton base and Cys1010 of C4b, are in red. (**C**) Coordinates from the model 3 simulation with no covalent bond between penton base and C4b. Cys1010 of C4b is in red. Penton base subunits are colored as in Figure 5. Penton base is shown in a different orientation for each simulation. The final coordinates from all three simulations show additional favorable interactions indicated with arrows between C4b and penton base chain D (yellow).

In order to test our hypothesis that C4b deposition on one RGD loop leads to the entanglement of C4b with additional RGD loops on the same penton base multimer, we built three starting models for molecular dynamics simulations. In model 1 the thioester of C4b is covalently bound to the hydroxyl group of Thr343 in one RGD loop (Figure 6A). In model 2, C4b is covalently bound to the amino group of Arg 347 in an RGD loop (Figure 6B). In model 3, we presume that the reactive thioester of C4b has reacted with a water molecule and position C4b near, but not covalently bound to, one RGD loop (Figure 6C). Using these three starting models we performed molecular dynamics simulations to observe whether nearby RGD loops would form favorable interactions with C4b. As noted by Flatt et al., the RGD loops move relatively quickly during molecular dynamics simulations, presumably because of their flexibility and intrinsic disorder [29]. We found that, within relatively short simulations (12 ns), stabilizing interactions formed between C4b and a nearby RGD loop (Figure 6).

Using the NAMD Energy plugin, we evaluated the stabilizing non-bonded interactions formed between C4b and each penton base RGD loop. In the model 1 simulation, C4b was covalently bound to a hydroxyl group in the RGD loop of the penton base chain C in the starting model. By the end of the simulation, favorable interactions had formed with RGD loops of two neighboring penton base subunits (chains C and D), with an overall strongly favorable interaction between C4b and penton base of −318 kcal/mol (Table 1). In the model 2 simulation, C4b was covalently bound to an amino group in the RGD loop of the penton base chain C in the starting model. Similar to the results of the model 1 simulation, by the end of the model 2 simulation, favorable interactions had formed with RGD loops of two neighboring penton base subunits (chains C and D). The calculated non-bonded interaction energy between C4b and penton base at the end of the model 2 simulation was even more favorable, −594 kcal/mol, (Table 2) than found for the model 1 simulation. In the model 3 simulation, C4b was positioned near the RGD loop of penton base chain C in the starting model. Similar to the results of the model 1 and model 2 simulations, by the end of the model 3 simulation, favorable interactions had formed with RGD loops of two neighboring penton base subunits (chains C and D). The non-bonded interaction energy between C4b and penton base at the end of the model 3 simulation, −315 kcal/mol (Table 3), was similar to that found for model 1. In each model simulation, the final non-bonded interaction energy was a combination of van der Waals (VdW) and electrostatic (Elec) components: model 1 (VdW: −132 kcal/mol; Elec: −186 kcal/mol), model 2 (VdW: −189 kcal/mol; Elec: −406 kcal/mol), and model 3 (VdW: −135 kcal/mol; Elec: −181 kcal/mol).

All three molecular dynamics simulations, presented in detail (models 1–3), support the idea that one molecule of C4b may form stabilizing interactions with multiple RGD loops of a HAdV-C5 penton base multimer. While a covalent bond between C4b and one RGD loop may promote the entanglement of multiple RGD loops (models 1 and 2), the model 3 simulation indicates that just positioning C4b near the penton base will also result in entanglement. We noted that, for all three models, secondary non-bonded interactions formed between C4b and the most extended RGD loop model of chain D (Figure 6, Table 1, Table 2 and Table 3). In contrast, preliminary models that did not have C4b positioned near the most extended RGD loop model (chain D) did not show the entanglement of RGD loops by the end of 12 ns simulations, and these models were rejected. Although the RGD loop is highly flexible, we did not observe the RGD loops of chains A, B, C, or E to extend as fully as that of chain D during the relatively short (12 ns) simulations. It is likely that, over longer simulations, all five RGD loops would extend and contract and that all five chains would be equally likely to interact with C4b. However, within the constraints of our analysis protocol, it seems that positioning C4b near an extended RGD loop is a critical factor for the acceptance of a C4b/penton base model. To confirm this idea, we built two additional starting models with covalent bonds between C4b and the penton base (models 4 and 5). These covalent linkages were made with residues within the RGD loops of chains C or E on either side of the most extended chain D RGD loop. Both models 4 and 5 showed entanglement with the chain D RGD loop by the end of a 12 ns simulation (Figures S1 and S2; Tables S1 and S2). One additional starting model without a covalent bond between C4b and penton base was generated (model 6), with C4b positioned over the chain E RGD loop. By the end of a 12 ns simulation, model 6 showed entanglement with the chain D RGD loop (Figure S3, Table S3). These additional C4b/penton base models (models 4–6) support the idea that positioning C4b near an extended RGD loop is a key factor for model acceptance with our simulation protocol. It seems likely that an abundant number of acceptable starting models could be generated that would result in the entanglement of RGD loops. The spacing of RGD loops at the top of the penton base (35 Å), the dimensions of the C4b thioester domain (~50 Å in diameter), and the multi-domain nature of C4b, all contribute to the likelihood of RGD loop interactions with C4b. In addition, longer molecular dynamics simulations would likely result in the observation of more RGD loop movement and an increase in C4b/RGD loop entanglement.

It has been proposed that integrin binding to the RGD loops of the penton base may induce a conformational change, or untwisting, of the penton base multimer that initiates AdV uncoating [34]. Together with the results presented in this study, it seems reasonable that the structural mechanism underlying the C4b neutralization of HAdV-C5 is the entanglement of C4b with multiple RGD loops of penton base multimers at each capsid vertex. This entanglement may lead to the stabilization of penton base multimers, which, in turn, may block capsid uncoating and prevent the release of the virally encapsidated endosomal membrane lytic factor, protein VI.

## 4. Discussion

The complement system has been described as keeping a constant vigil against viruses [48]. This system has an ancient origin, existing in a primitive form in a “living fossil”, the horseshoe crab (*Carcinoscorpius rotundicauda*) [49]. In humans, a proteolytic cascade of multiple complement proteins serves to detect and mark viruses and other pathogens for destruction. The interaction of either multiple IgG molecules or a single IgM molecule with an AdV virion can initiate the classical complement activation pathway. Bottermann et al. have shown that neutralizing antibodies act with complement components C1 and C4 to effect AdV neutralization by blocking the release of AdV/C4b complexes from the endosome [24]. They also showed that this complement-based antiviral pathway works in parallel with the tripartite motif-containing protein 21 (TRIM21) antiviral activity [50]. TRIM21 is an intracellular antibody receptor that triggers the proteosome-dependent degradation of antibody-virus complexes that enter the cytoplasm [51].

In this computational modeling study, we investigated the possibility that C4b might neutralize HAdV-C5 by binding and entangling the flexible penton base RGD loops at the capsid vertices. We reasoned that the entanglement of multiple RGD loops at the same vertex might stabilize the penton base and block the conformational changes needed for the release of the penton base and the membrane lytic factor protein VI. We used available structural information for HAdV-C5 [36], HAdV-C5 anti-hexon antibody 9C12 complexes [32,33], a cryo-EM structure of an IgG-C1 complex [22], and a cryo-ET structure of an IgM-C1-C4 complex [19], to build a composite HAdV-C5/9C12/C1/C4b model (Figure 4B). This model positions C4b over the penton base capsomers of the HAdV-C5 capsid with the C4b reactive thioester positioned near the intrinsically disordered RGD loops of the penton base. Our molecular dynamics simulations with C4b and penton base indicate that it is possible for C4b to interact with multiple RGD loops at the same vertex (Figure 6) and that favorable non-bonded interactions may be formed that could stabilize the penton base (Table 1, Table 2 and Table 3) and thus block capsid uncoating. The molecular dynamics results support our hypothesis that C4b neutralizes HAdV-C5 by stabilizing penton base capsomers via RGD loop entanglement. Thus, the intrinsically disordered RGD loops of the HAdV-C5 penton base may provide a functional advantage for interacting with αv integrins on host cells, while at the same time serving as an Achilles heel of the virus, which can be exploited by the complement system.

In building a model for 9C12 Fab fragments interacting with the HAdV-C5 capsid (Figure 1D), we observed a steric clash between Fab arms bound at neighboring peripentonal hexons (Figure 2). We reasoned that this steric clash might be resolved with conformational changes of the hexon epitopes or within the IgG molecule. In fact, the observation of a steric clash at this position is consistent with past observations. The cryo-EM structure of the HAdV-C5/9C12 complex indicated that 9C12 binds bivalently to neighboring peripentonal hexons with a distorted conformation for one of the two Fab arms [33]. Bottermann et al. found that 9C12 has a slow on-rate and that binding occurs with a concurrent cost in entropy [32]. In light of our current structural modeling study, it seems reasonable to propose that distorted, bivalently bound 9C12 IgGs at the peripentonal hexons might initiate C1 binding in the vertex region of HAdV-C5. It is known that the C1q binding sites on IgGs are normally shielded by the Fab arms and are generally only exposed after the binding of IgG to a pathogen [43]. The antihexon 9C12 IgG may be able to efficiently neutralize HAdV-C5 via C4b opsonization by virtue of its favorable epitope position and its distorted binding mode, which likely exposes C1q binding sites near the IgG hinge region. The C4b mediated neutralization of HAdV-C5 may be enhanced by the preferential binding of 9C12 to peripentonal hexons versus hexons in the middle of the facet [33]. Our model of the HAdV-C5/9C12/C1/C4b complex indicates that the preferential binding of 9C12 to peripentonal hexons would ensure that a good percentage of C4b would opsonize the virus in the vicinity of the penton base.

Interestingly, our structural modeling study may offer a possible explanation for why the minimum ratio of 9C12 to HAdV-C5 for neutralization is 240 antibody molecules per virus particle [33]. We propose that the complement-mediated neutralization mechanism of 9C12 is the recruitment of C1 and C4b to the vertex regions, the entanglement of the penton base RGD loops by C4b, and the stabilization of the penton base, which leads to the blockage of capsid uncoating steps, including the release of protein VI. If we are correct, then it would be important to stabilize all twelve of the penton base capsomers on a particular virus particle to achieve neutralization by the complement-based pathway. In other words, the binding of C4b to only a subset of the penton base capsomers would not be expected to completely block protein VI release and HAdV-C5 would not be neutralized by this antiviral pathway. Our model of the HAdV-C5/9C12 complex suggests that a ratio of 240 antibody molecules per virus particle would lead to the bivalent binding of 9C12 at all peripentonal hexons, as well as a sufficient number of IgG molecules bound in the middle of each facet to promote the formation of F_C_ platforms near the peripentonal hexons. These well-positioned F_C_ platforms would effectively prime the system for the recruitment of C1 and C4b to the vicinity of the capsid vertices and heighten the chances for stabilization of all twelve of the penton base capsomers.

A limitation of our study is that the HAdV-C5 fiber protein was not included in our model of the HAdV-C5/9C12/C1/C4b complex. Multiple studies indicate that fiber is released during early AdV cell entry steps, which occur at the plasma membrane [52]. Therefore, we reasoned that the neutralization mechanism of C4b was likely not dependent on the presence of fiber. If, however, the fiber is still present when C4b is opsonizing the HAdV-C5/9C12/C1 complex, then we would anticipate that the fiber would present additional possible opsonization sites and further opportunities for C4b to form stabilizing non-bonded interactions with HAdV-C5 capsid proteins. Indeed, Bottermann et al. observed that C4b deposition on the viral capsid interferes with fiber and penton base shedding during in vitro heat treatment assays [24]. The entanglement of both the fiber and penton base RGD loops by C4b is an alternative and plausible neutralization mechanism. Nevertheless, the molecular dynamics simulations presented in this study indicate that if C4b is positioned near a HAdV-C5 vertex, then the entanglement of penton base RGD loops and stabilization of the multimeric penton base capsomer are likely outcomes (Figure 6). Previous cryo-EM studies of AdV/integrin complexes suggest that symmetry mismatched interactions between integrins and the penton base trigger the untwisting of the penton base pentamers and the release of the penton base from the capsid [34]. We envision that the C4b entanglement of the RGD loops would have the opposite effect and serve to lock penton the base capsomers firmly in the AdV capsid.

This work suggests that introducing mutations into the penton base RGD loops might be a feasible strategy to modulate the interaction of HAdV-C5 with the complement system. However, simply mutating the serine, threonine, lysine and arginine residues in the RGD loops to other residue types might not be sufficient, as our study indicates that C4b can entangle multiple RGD loops even without being covalently bound to the penton base. Analysis of the non-bonded interaction energies formed between RGD loops and C4b during molecular dynamics simulations indicates that both sizable van der Waals and electrostatic interactions are formed (Table 1, Table 2 and Table 3). Strategies to minimize the C4b entanglement of the penton base RGD loops might include shortening the RGD loops to diminish van der Waals interactions, and reducing the number of charged residues in the RGD loops to minimize possible electrostatic interactions with C4b. These proposed modification strategies would require experimental testing and verification. It is of interest to note that the vast majority of HAdV species have very short penton base RGD loops that may point to an evolutionary complement attack evasion mechanism [48,53]. Whether or not HAdV species with short penton base RGD loops are more resistant to C4 complement-mediated neutralization, compared to HAdv species C, also requires experimental verification. In addition, RGD loop modifications would have to be designed so that either they do not impair interactions with αv integrins on host cells or so that they provide targeting to alternative internalization receptors. Atasheva et al. have demonstrated that it is possible to replace HAdV-C5 RGD loops with sequences derived from human laminin-α1 to retarget the virus to use α3β1, α6β1, and α6β4 integrins present on human epithelial tumor cells [11].

A potential limitation of our study is that our analysis and conclusions are based exclusively on computational modeling of AdV interactions with antibodies and complement components C1q and C4. As the detrimental effect of neutralizing antibodies and complement on the safety and efficacy of AdV-based vectors has been extensively reported, numerous approaches to shield AdV particles from blood factors have been proposed. Many of these approaches have been tested in pre-clinical models and in human clinical trials, including shielding AdV with polymers [54] and plasma proteins, such as albumin [55]. Our study may serve as a foundation for engineering novel AdV vectors that resist complement-mediated inactivation based on specific targeted mutations in the adenovirus hexon and penton base. While our study is purely theoretical, the analyses we have done point to the conceptual feasibility of designing AdV vectors resistant to C4 complement deposition on penton base capsomers. It is certain that the direct visualization of AdV in complex with complement components, using cryo-EM or cryo-ET approaches, will be required to provide exhaustive information on the mode of complement-AdV interaction. It is anticipated that these structural studies would lead to the design and experimental validation of mutant AdV variants with improved resistance to complement-mediated neutralization.

Together, our modeling and molecular dynamics results provide a structural hypothesis for complement C4 mediated neutralization of AdV. An enhanced understanding of the molecular mechanisms underlying the interaction of AdV with host factors, including complement proteins, should promote the development of AdV-based oncolytic viruses and gene therapy vectors.

## Figures and Tables

**Table 1:** Total non-bonded interaction energy between C4b and penton base for model 1 with covalent linkage to penton base Thr343.

	0 ns (kcal/mol)	12 ns (kcal/mol)
Penton base Chain A (pink)	0	0
Penton base Chain B (blue)	0	0
Penton base Chain C (green)	−51	−226
Penton base Chain D (yellow)	6	−91
Penton base Chain E (orange)	0	0
Penton base Chains A–E	−45	−318

**Table 2:** Total non-bonded interaction energy between C4b and penton base for model 2 with covalent linkage to penton base Arg347.

	0 ns (kcal/mol)	12 ns (kcal/mol)
Penton base Chain A (pink)	0	0
Penton base Chain B (blue)	4	−10
Penton base Chain C (green)	−121	−517
Penton base Chain D (yellow)	1	−67
Penton base Chain E (orange)	0	0
Penton base Chains A–E	−116	−594

**Table 3:** Total non-bonded interaction energy between C4b and penton base for model 3 with no covalent linkage to penton base.

	0 ns (kcal/mol)	12 ns (kcal/mol)
Penton base Chain A (pink)	0	0
Penton base Chain B (blue)	0	29
Penton base Chain C (green)	−61	−158
Penton base Chain D (yellow)	0	−186
Penton base Chain E (orange)	0	0
Penton base Chains A–E	−61	−315

## Data Availability

The models presented in this study are available on request from the corresponding author.

## Supplementary Materials

The following are available online at https://www.mdpi.com/1999-4915/13/1/111/s1, Figure S1: Interaction of C4b with multiple RGD loops of HAdV-C5 penton base for Model 4; Figure S2: Interaction of C4b with multiple RGD loops of HAdV-C5 penton base for Model 5; Figure S3: Interaction of C4b with multiple RGD loops of HAdV-C5 penton base for Model 6; Table S1: Total Non-bonded Interaction Energy between C4b and Penton Base for Model 4; Table S2: Total Non-bonded Interaction Energy between C4b and Penton Base for Model 5; Table S3: Total Non-bonded Interaction Energy between C4b and Penton Base for Model 6.

## References

[1] Niemann J., Woller N., Brooks J., Fleischmann-Mundt B., Martin N.T., Kloos A., Knocke S., Ernst A.M., Manns M.P., Kubicka S. (2019). Molecular retargeting of antibodies converts immune defense against oncolytic viruses into cancer immunotherapy. Nat. Commun..

[2] Allen R.J., Byrnes A.P. (2019). Interaction of adenovirus with antibodies, complement, and coagulation factors. FEBS Lett..

[3] Shirley J.L., de Jong Y.P., Terhorst C., Herzog R.W. (2020). Immune Responses to Viral Gene Therapy Vectors. Mol. Ther..

[4] Roberts D.M., Nanda A., Havenga M.J., Abbink P., Lynch D.M., Ewald B.A., Liu J., Thorner A.R., Swanson P.E., Gorgone D.A. (2006). Hexon-chimaeric adenovirus serotype 5 vectors circumvent pre-existing anti-vector immunity. Nature.

[5] Zhu F.C., Li Y.H., Guan X.H., Hou L.H., Wang W.J., Li J.X., Wu S.P., Wang B.S., Wang Z., Wang L. (2020). Safety, tolerability, and immunogenicity of a recombinant adenovirus type-5 vectored COVID-19 vaccine: A dose-escalation, open-label, non-randomised, first-in-human trial. Lancet.

[6] Ferguson M.S., Lemoine N.R., Wang Y. (2012). Systemic delivery of oncolytic viruses: Hopes and hurdles. Adv. Virol..

[7] Khare R., Hillestad M.L., Xu Z., Byrnes A.P., Barry M.A. (2013). Circulating antibodies and macrophages as modulators of adenovirus pharmacology. J. Virol..

[8] Xu Z., Tian J., Smith J.S., Byrnes A.P. (2008). Clearance of adenovirus by Kupffer cells is mediated by scavenger receptors, natural antibodies, and complement. J. Virol..

[9] Kalyuzhniy O., Di Paolo N.C., Silvestry M., Hofherr S.E., Barry M.A., Stewart P.L., Shayakhmetov D.M. (2008). Adenovirus serotype 5 hexon is critical for virus infection of hepatocytes in vivo. Proc. Natl. Acad. Sci. USA.

[10] Waddington S.N., McVey J.H., Bhella D., Parker A.L., Barker K., Atoda H., Pink R., Buckley S.M., Greig J.A., Denby L. (2008). Adenovirus serotype 5 hexon mediates liver gene transfer. Cell.

[11] Atasheva S., Emerson C.C., Yao J., Young C., Stewart P.L., Shayakhmetov D.M. (2020). Systemic cancer therapy with engineered adenovirus that evades innate immunity. Sci. Transl. Med..

[12] Xu Z., Qiu Q., Tian J., Smith J.S., Conenello G.M., Morita T., Byrnes A.P. (2013). Coagulation factor X shields adenovirus type 5 from attack by natural antibodies and complement. Nat. Med..

[13] Doronin K., Flatt J.W., Di Paolo N.C., Khare R., Kalyuzhniy O., Acchione M., Sumida J.P., Ohto U., Shimizu T., Akashi-Takamura S. (2012). Coagulation factor X activates innate immunity to human species C adenovirus. Science.

[14] Newton A.H., Cardani A., Braciale T.J. (2016). The host immune response in respiratory virus infection: Balancing virus clearance and immunopathology. Semin. Immunopathol..

[15] Barnum S.R. (2017). Complement: A primer for the coming therapeutic revolution. Pharm. Ther..

[16] Merle N.S., Church S.E., Fremeaux-Bacchi V., Roumenina L.T. (2015). Complement System Part I—Molecular Mechanisms of Activation and Regulation. Front. Immunol..

[17] Burton D.R. (1986). Is IgM-like dislocation a common feature of antibody function?. Immunol. Today.

[18] Feinstein A., Munn E.A. (1969). Conformation of the free and antigen-bound IgM antibody molecules. Nature.

[19] Sharp T.H., Boyle A.L., Diebolder C.A., Kros A., Koster A.J., Gros P. (2019). Insights into IgM-mediated complement activation based on in situ structures of IgM-C1-C4b. Proc. Natl. Acad. Sci. USA.

[20] de Jong R.N., Beurskens F.J., Verploegen S., Strumane K., van Kampen M.D., Voorhorst M., Horstman W., Engelberts P.J., Oostindie S.C., Wang G. (2016). A Novel Platform for the Potentiation of Therapeutic Antibodies Based on Antigen-Dependent Formation of IgG Hexamers at the Cell Surface. PLoS Biol..

[21] Diebolder C.A., Beurskens F.J., de Jong R.N., Koning R.I., Strumane K., Lindorfer M.A., Voorhorst M., Ugurlar D., Rosati S., Heck A.J. (2014). Complement is activated by IgG hexamers assembled at the cell surface. Science.

[22] Ugurlar D., Howes S.C., de Kreuk B.J., Koning R.I., de Jong R.N., Beurskens F.J., Schuurman J., Koster A.J., Sharp T.H., Parren P. (2018). Structures of C1-IgG1 provide insights into how danger pattern recognition activates complement. Science.

[23] Reid K.B., Porter R.R. (1976). Subunit composition and structure of subcomponent C1q of the first component of human complement. Biochem. J..

[24] Bottermann M., Foss S., Caddy S.L., Clift D., van Tienen L.M., Vaysburd M., Cruickshank J., O’Connell K., Clark J., Mayes K. (2019). Complement C4 Prevents Viral Infection through Capsid Inactivation. Cell Host Microbe.

[25] Maier O., Marvin S.A., Wodrich H., Campbell E.M., Wiethoff C.M. (2012). Spatiotemporal dynamics of adenovirus membrane rupture and endosomal escape. J. Virol..

[26] Wiethoff C.M., Wodrich H., Gerace L., Nemerow G.R. (2005). Adenovirus protein VI mediates membrane disruption following capsid disassembly. J. Virol..

[27] Ganz T. (2003). Defensins: Antimicrobial peptides of innate immunity. Nat. Rev. Immunol..

[28] Smith J.G., Nemerow G.R. (2008). Mechanism of adenovirus neutralization by Human alpha-defensins. Cell Host Microbe.

[29] Flatt J.W., Kim R., Smith J.G., Nemerow G.R., Stewart P.L. (2013). An intrinsically disordered region of the adenovirus capsid is implicated in neutralization by human alpha defensin 5. PLoS ONE.

[30] Gaboriaud C., Juanhuix J., Gruez A., Lacroix M., Darnault C., Pignol D., Verger D., Fontecilla-Camps J.C., Arlaud G.J. (2003). The crystal structure of the globular head of complement protein C1q provides a basis for its versatile recognition properties. J. Biol. Chem..

[31] Saphire E.O., Parren P.W., Pantophlet R., Zwick M.B., Morris G.M., Rudd P.M., Dwek R.A., Stanfield R.L., Burton D.R., Wilson I.A. (2001). Crystal structure of a neutralizing human IGG against HIV-1: A template for vaccine design. Science.

[32] Bottermann M., Lode H.E., Watkinson R.E., Foss S., Sandlie I., Andersen J.T., James L.C. (2016). Antibody-antigen kinetics constrain intracellular humoral immunity. Sci. Rep..

[33] Varghese R., Mikyas Y., Stewart P.L., Ralston R. (2004). Postentry neutralization of adenovirus type 5 by an antihexon antibody. J. Virol..

[34] Lindert S., Silvestry M., Mullen T.M., Nemerow G.R., Stewart P.L. (2009). Cryo-electron microscopy structure of an adenovirus-integrin complex indicates conformational changes in both penton base and integrin. J. Virol..

[35] Wickham T.J., Mathias P., Cheresh D.A., Nemerow G.R. (1993). Integrins alpha v beta 3 and alpha v beta 5 promote adenovirus internalization but not virus attachment. Cell.

[36] Dai X., Wu L., Sun R., Zhou Z.H. (2017). Atomic Structures of Minor Proteins VI and VII in Human Adenovirus. J. Virol..

[37] Pettersen E.F., Goddard T.D., Huang C.C., Meng E.C., Couch G.S., Croll T.I., Morris J.H., Ferrin T.E. (2021). UCSF ChimeraX: Structure visualization for researchers, educators, and developers. Protein Sci..

[38] Pettersen E.F., Goddard T.D., Huang C.C., Couch G.S., Greenblatt D.M., Meng E.C., Ferrin T.E. (2004). UCSF Chimera—A visualization system for exploratory research and analysis. J. Comput. Chem..

[39] Mortensen S., Kidmose R.T., Petersen S.V., Szilagyi A., Prohaszka Z., Andersen G.R. (2015). Structural Basis for the Function of Complement Component C4 within the Classical and Lectin Pathways of Complement. J. Immunol..

[40] Phillips J.C., Hardy D.J., Maia J.D.C., Stone J.E., Ribeiro J.V., Bernardi R.C., Buch R., Fiorin G., Henin J., Jiang W. (2020). Scalable molecular dynamics on CPU and GPU architectures with NAMD. J. Chem. Phys..

[41] Best R.B., Zhu X., Shim J., Lopes P.E., Mittal J., Feig M., Mackerell A.D. (2012). Optimization of the additive CHARMM all-atom protein force field targeting improved sampling of the backbone phi, psi and side-chain chi (1) and chi (2) dihedral angles. J. Chem. Theory Comput..

[42] Humphrey W., Dalke A., Schulten K. (1996). VMD: Visual molecular dynamics. J. Mol. Graph..

[43] Vidarsson G., Dekkers G., Rispens T. (2014). IgG subclasses and allotypes: From structure to effector functions. Front. Immunol..

[44] Zhang X., Zhang L., Tong H., Peng B., Rames M.J., Zhang S., Ren G. (2015). 3D Structural Fluctuation of IgG1 Antibody Revealed by Individual Particle Electron Tomography. Sci. Rep..

[45] Wang G., de Jong R.N., van den Bremer E.T., Beurskens F.J., Labrijn A.F., Ugurlar D., Gros P., Schuurman J., Parren P.W., Heck A.J. (2016). Molecular Basis of Assembly and Activation of Complement Component C1 in Complex with Immunoglobulin G1 and Antigen. Mol. Cell.

[46] Law S.K. (1983). The covalent binding reaction of C3 and C4. Ann. N. Y. Acad. Sci.

[47] Zubieta C., Schoehn G., Chroboczek J., Cusack S. (2005). The structure of the human adenovirus 2 penton. Mol. Cell.

[48] Agrawal P., Nawadkar R., Ojha H., Kumar J., Sahu A. (2017). Complement Evasion Strategies of Viruses: An Overview. Front. Microbiol..

[49] Zhu Y., Thangamani S., Ho B., Ding J.L. (2005). The ancient origin of the complement system. EMBO J..

[50] Mallery D.L., McEwan W.A., Bidgood S.R., Towers G.J., Johnson C.M., James L.C. (2010). Antibodies mediate intracellular immunity through tripartite motif-containing 21 (TRIM21). Proc. Natl. Acad. Sci. USA.

[51] Foss S., Watkinson R., Sandlie I., James L.C., Andersen J.T. (2015). TRIM21: A cytosolic Fc receptor with broad antibody isotype specificity. Immunol. Rev..

[52] Greber U.F., Flatt J.W. (2019). Adenovirus Entry: From Infection to Immunity. Annu. Rev. Virol..

[53] Mellors J., Tipton T., Longet S., Carroll M. (2020). Viral Evasion of the Complement System and Its Importance for Vaccines and Therapeutics. Front. Immunol..

[54] Wu Y., Li L., Frank L., Wagner J., Andreozzi P., Hammer B., D’Alicarnasso M., Pelliccia M., Liu W., Chakrabortty S. (2019). Patchy Amphiphilic Dendrimers Bind Adenovirus and Control Its Host Interactions and in Vivo Distribution. ACS Nano.

[55] Rojas L.A., Condezo G.N., Moreno R., Fajardo C.A., Arias-Badia M., San Martin C., Alemany R. (2016). Albumin-binding adenoviruses circumvent pre-existing neutralizing antibodies upon systemic delivery. J. Control. Release.

