# Supplementary material for: Structure-Based Modeling of Complement C4 Mediated Neutralization of Adenovirus
Source: Viruses. 2021 Jan 15;13(1):111. doi: 10.3390/v13010111 (PMC7830055; doi:10.3390/v13010111)
Supplement: Supplementary file 1 [file viruses-13-00111-s001.pdf]

SUPPLEMENTARY MATERIALS

For

Structure-based Modeling of Complement C4 Mediated Neutralization of Adenovirus

Corey C. Emerson[1] and Phoebe L. Stewart[1,2]

1. Department of Pharmacology and Cleveland Center for Membrane and Structural Biology, Case Western Reserve University, Cleveland, OH 44106 USA

*Correspondence: pls47@case.edu

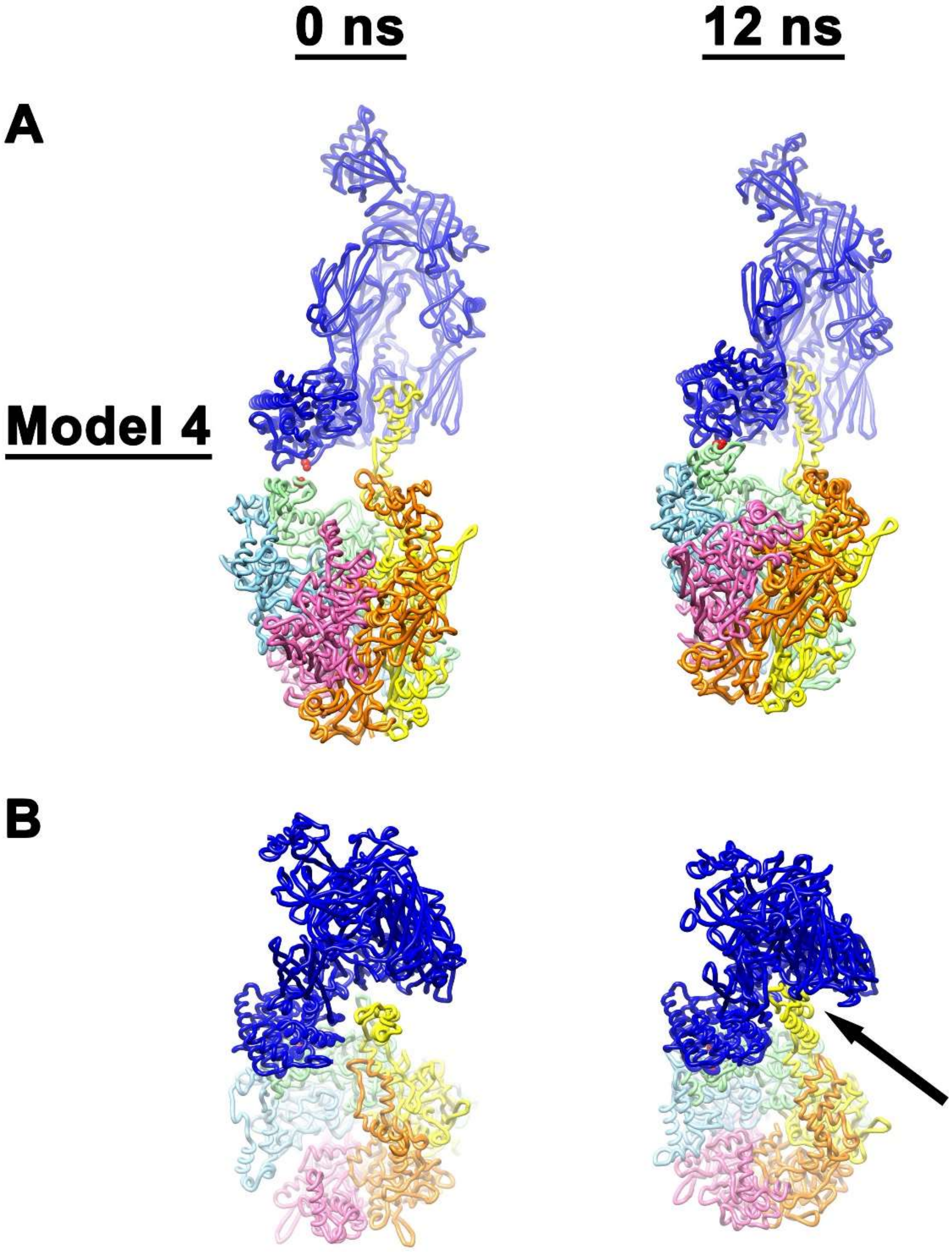

Figure S1. Interaction of C4b with multiple RGD loops of HAdV-C5 penton base for Model 4. A) Initial (0 ns) and final (12 ns) coordinates from the Model 4 molecular dynamics simulation with a covalent bond between Thr346 of penton base chain C (green) and C4b (blue) shown in side view. B) Top view of initial and final coordinates. The covalently linked residues, Thr346 of penton base and Cys1010 of C4b, are in red. Penton base subunits are colored as follows: chain A, pink; chain B, light blue; chain C, green; chain D, yellow; and chain E, orange. The final coordinates show additional favorable interactions indicated with an arrow between C4b and penton base chain D (yellow).

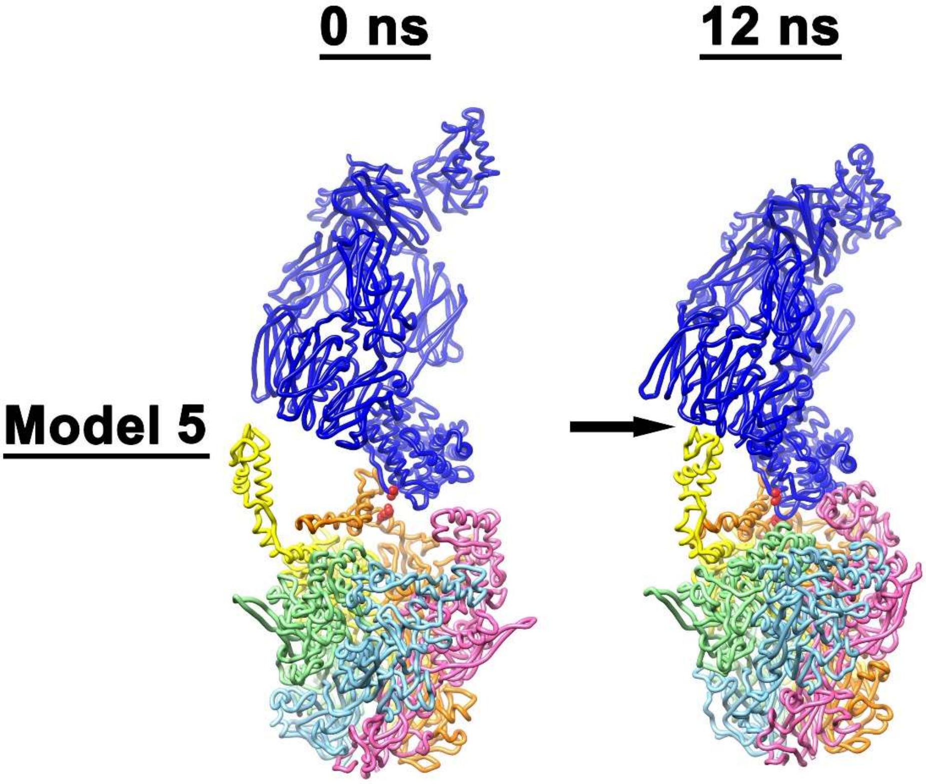

Figure S2. Interaction of C4b with multiple RGD loops of HAdV-C5 penton base for Model 5. Initial (0 ns) and final (12 ns) coordinates from the Model 5 molecular dynamics simulation with a covalent bond between Lys297 of penton base chain E (orange) and C4b. The covalently linked residues, Lys297 of penton base and Cys1010 of C4b, are in red. Penton base subunits are colored as in Figure S1. The final coordinates show additional favorable interactions indicated with an arrow between C4b and penton base chain D (yellow).

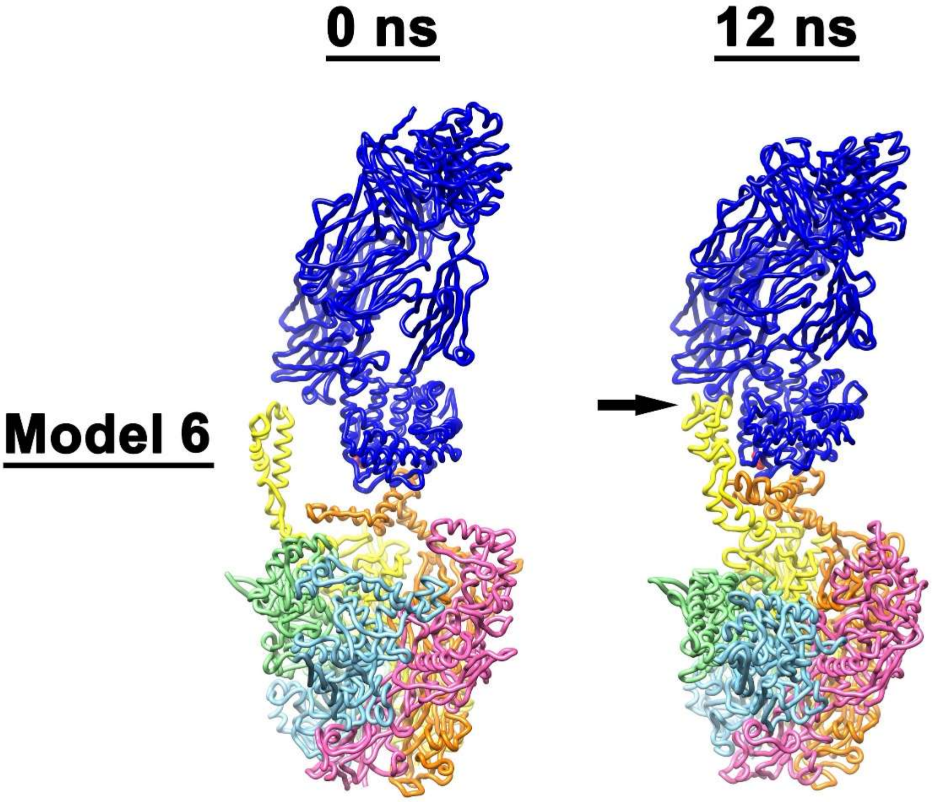

Figure S3. Interaction of C4b with multiple RGD loops of HAdV-C5 penton base for Model 6. Initial (0 ns) and final (12 ns) coordinates from the Model 6 molecular dynamics simulation with no covalent bond between penton base and C4b. Cys1010 of C4b is in red. Penton base subunits are colored as in Figure S1. The final coordinates show additional favorable interactions indicated with an arrow between C4b and penton base chain D (yellow).

Table S1. Total Non-bonded Interaction Energy between C4b and Penton Base for Model 4

	0 ns (kcal/mol)	12 ns (kcal/mol)
Penton base Chain A (pink)	0	0
Penton base Chain B (blue)	0	5
Penton base Chain C (green)	-31	-373
Penton base Chain D (yellow)	-17	-454
Penton base Chain E (orange)	1	0
**Penton base Chains A-E**	**-47**	**-822**

Table S2. Total Non-bonded Interaction Energy between C4b and Penton Base for Model 5

	0 ns (kcal/mol)	12 ns (kcal/mol)
Penton base Chain A (pink)	0	3
Penton base Chain B (blue)	0	0
Penton base Chain C (green)	0	0
Penton base Chain D (yellow)	1	-35
Penton base Chain E (orange)	-48	-161
**Penton base Chains A-E**	**-47**	**-193**

Table S3. Total Non-bonded Interaction Energy between C4b and Penton Base for Model 6

	0 ns (kcal/mol)	12 ns (kcal/mol)
Penton base Chain A (pink)	0	0
Penton base Chain B (blue)	0	0
Penton base Chain C (green)	0	0
Penton base Chain D (yellow)	0	-484
Penton base Chain E (orange)	-70	-254
**Penton base Chains A-E**	**-70**	**-738**
